# Factors Contributing to the Utilization of Adult Mental Health Services in Children and Adolescents Diagnosed with Hyperkinetic Disorder

**DOI:** 10.1100/2012/451205

**Published:** 2012-04-30

**Authors:** Hilario Blasco-Fontecilla, Juan J. Carballo, Rebeca Garcia-Nieto, Jorge Lopez-Castroman, Analucia A. Alegria, Ignacio Basurte-Villamor, Juncal Sevilla-Vicente, Rocio Navarro-Jimenez, Teresa Legido-Gil, Consuelo Morant-Ginestar, Miguel Angel Jimenez-Arriero, Jeronimo Saiz-Ruiz, Enrique Baca-Garcia

**Affiliations:** ^1^Department of Psychiatry. IIS-Fundación Jiménez Díaz, Autonoma University, 28040 Madrid, Spain; ^2^Centro de Investigación Biomédica en Red (CIBERSAM), 28007 Madrid, Spain; ^3^Departament of Psiquiatry, Hospital Ramón y Cajal, Alcalá University, 28034 Madrid, Spain; ^4^Department of Mental Health, Madrid Regional Health Council, 28013 Madrid, Spain; ^5^Department of Psychiatry, Hospital Doce de Octubre, Complutense University, 28041 Madrid, Spain; ^6^Department of Psychiatry, New York State Psychiatric Institute, Columbia University, New York, NY 10032, USA

## Abstract

*Objectives*. To examine whether age of First diagnosis, gender, psychiatric comorbidity, and treatment modalities (pharmacotherapy or psychotherapy) at Child and Adolescent Mental Health Services (CAMHS) moderate the risk of Adult Mental Health Services (AMHS) utilization in patients diagnosed with hyperkinetic disorder at CAMHS. *Methods*. Data were derived from the Madrid Psychiatric Cumulative Register Study. The target population comprised 32,183 patients who had 3 or more visits at CAMHS. Kaplan-Meier curves were used to assess survival data. A series of logistic regression analyses were performed to study the role of age of diagnosis, gender, psychiatric comorbidity, and treatment modalities. *Results*. 7.1% of patients presented with hyperkinetic disorder at CAMHS. Compared to preschool children, children and adolescents first diagnosed with hyperkinetic disorder at CAMHS were more likely to use AMHS. Female gender and comorbidity with affective disorders, schizophrenia, schizotypal and delusional disorders increased the risk of use of AMHS. Pharmacological or combined treatment of hyperkinetic disorder diagnosed at CAMHS was associated with increased risk of use at AMHS. *Conclusions*. Older age of first diagnosis, female gender, psychiatric comorbidity, and pharmacological treatment at CAMHS are markers of risk for the transition from CAMHS to AMHS in patients with hyperkinetic disorder diagnosed at CAMHS.

## 1. Introduction

Research regarding mental health services use is of significant interest given the ongoing efforts to control rising costs of mental health care. Thus, factors related to the transition from Child and Adolescent Mental Health Services (CAMHSs) to Adult Mental Health Services (AMHSs) are a major concern [1]. Longitudinal data assessing continuities of childhood disorders into adulthood [2] may allow to implement early intervention and preventive programmes [3], thus reducing the economic burden of childhood mental disorders progressing into adulthood.

In this context, the study of the continuities of either attention-deficit/hyperactivity disorder (ADHD, DSM-IV), a condition affecting between 4% and 8% of children worldwide [4–6], or hyperkinetic disorder (HD) (ICD-10), with a 1% worldwide prevalence rate, appears to be relevant [7]. Longitudinal studies followingup children with ADHD have consistently reported high rates of ADHD at follow up [7, 8]. The rate of syndromatic remission of ADHD is around 60% [9, 10]. The majority of subjects, however, continue to struggle with a large number of ADHD symptoms in adulthood [9]. Unfortunately, there is little information about which factors could be associated with an increased risk of utilization of AMHS in patients diagnosed with HD at CAMHS. For instance, it is difficult to determine if age and gender are factors that may increase the risk of utilization of AMHS. Although HD is less frequent in adolescents compared with children [11], continuities of mental disorders are stronger for juvenile diagnosis than for diagnosis made prior to adolescence [12]. Regarding gender, although the clinical profile is similar in boys and girls [13], HD is less prevalent among girls [11, 14, 15]. *A priori*, severity of symptoms and psychiatric comorbidity are predictors of ADHD persistence [16] and, therefore, should intuitively be associated with an increased use of AMHS. Unfortunately, the bulk of studies have not addressed whether comorbidity moderates treatment outcomes in children with hyperactivity [17]. Finally, pharmacological treatment of HD could intuitively be considered a marker of poor prognosis, as children with HD under pharmacological treatment usually display a more severe clinical profile than children with HD treated with psychotherapy. However, pharmacological treatment could also improve HD, thus preventing the transition from CAMHS to AMHS.

The main purpose of this epidemiological study was to examine whether age of first diagnosis at CAMHS, gender, psychiatric comorbidity diagnosed at CAMHS, and treatment modalities (pharmacotherapy or psychotherapy) at CAMHS moderate the risk of AMHS utilization in patients diagnosed with HD at CAMHS.

## 2. Methods

Data were derived from the Madrid Psychiatric Cumulative Register (MCR) Study. The MCR study has been described in detail elsewhere [18–20]. Briefly, the MCR study is a naturalistic study of diagnostic stability over time. Between January 1986 and December 2007, public mental health centers in the province of Madrid, Spain, recorded all psychiatric visits in a registry. Nonstandardized clinical diagnoses and type of care provided were registered in every follow-up visit by experienced psychiatrists and clinical psychologists. All subjects can be traced because each patient is given a unique, anonymous identifying number, which is the same throughout all contacts with mental health services within the study area.

### 2.1. Diagnostic Procedure

Lifetime psychiatric diagnoses were made according to the International Classification of Diseases, Tenth Revision (ICD-10, World Health Organization, 1992). The diagnosis of either HD or any other mental health disorder was established after three consecutive visits within the same episode at CAMHS.

### 2.2. Participants

The target population comprised 32,183 children and adolescents (50.3% females and 49.7% males) who had 3 or more consecutive visits at CAMHS and were 18 years old or older at the time of the present study. We classified patients into four groups according to their first contact with CAMHS and using the US National Library of Medicine and the National Institutes of Health Classification: from birth to 23 months (HD infant group), from 2 to 5 years old (HD preschool children group), from 6 to 12 years old (HD children group), and from 13 to 17 years old (HD adolescent group).

### 2.3. Predictors of Treatment Use at AMHS

We examined three types of predictors of treatment use at AMHS among individuals diagnosed with HD during childhood and adolescence: sociodemographic characteristics (age of first diagnosis, gender), psychiatric comorbidity, and treatment modalities during followup at CAMHS.

### 2.4. Data Analyses

First, we used Kaplan-Meier survival curves to compare the likelihood of AMHS use among subjects diagnosed with and without HD at CAMHS. Among individuals with a diagnosis of HD during childhood or adolescence, a series of logistic regression analyses were subsequently performed to examine whether age of first psychiatric diagnosis, gender, and the presence of comorbid psychiatric disorders during followup at CAMHS increased the risk of AMHS use. We calculated odds ratios (ORs) and 95% confidence intervals (CIs) for ease of interpretation. Statistical significance was evaluated at the  .05 level using two-tailed tests. Finally, we used Fisher's Exact Test (FET) to evaluate the role of treatment (pharmacotherapy, psychotherapy, or both) regarding the transition from CAMHS to AMHS. We used SPSS statistical software, edition 17.0 for Mac (2008) for all statistical analyses.

## 3. Results

### 3.1. Sample Characteristics

Patients averaged 20 lifetime visits (SD ± 28, range: 4–1036). At the time of first evaluation at CAMHS, 0.1% were infants, 4.4% preschool children, 33.9% children, and 61.7% adolescents. 7.1% of the 32,183 patients were diagnosed with HD before adulthood (0.2% diagnosed as infants, 11.5% as preschool children, 60.0% as children, and 28.2% as adolescents).

### 3.2. Utilization of AMHS

**Figure 1:**
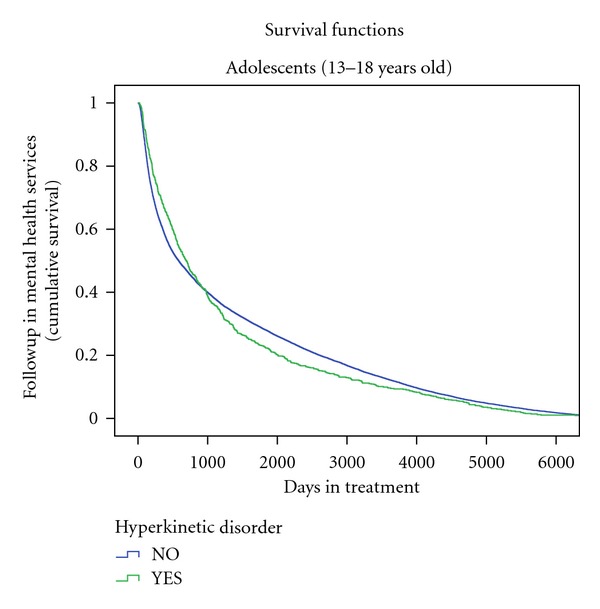
Kaplan-Meier curve of adolescents (13–18 years old) diagnosed with and without Hyperkinetic disorder. Cumulative survival decreased steadily during the followup in both subjects diagnosed with and without HD at CAMHS, but the decrease was more evident in those subjects with HD. The transition from CAMHS to AMHS takes place within a 1,830 day (5 years) period.

Subjects diagnosed with HD at CAMHS (*n* = 2, 274) were less likely to be followedup at AMHS (*n* = 425, 18.7%) than subjects without a diagnosis of HD at CAMHS (*n* = 10796, 36.1%) (OR (CI 95%) = 0.43 (0.38–0.48); FET *P* < 0.001). (see Figure 1).

Children and particularly adolescents first diagnosed with HD at CAMHS were more likely to seek mental health treatment at AMHS than the HD preschool children group (see Table 1). In addition, girls diagnosed with HD at CAMHS used AMHS more frequently than boys diagnosed with HD at CAMHS. Interestingly, subjects diagnosed with HD at CAMHS were less likely to present a comorbid diagnosis of schizophrenia, schizotypal and delusional disorders (F20–29) (OR = 0.585, 95%  CI = 0.458–0.747), or affective disorders (F30–39) (OR = 0.449, 95%  CI = 0.376–0.536) during followup at CAMHS. However, comorbidity of HD with any of the above-mentioned disorders (either F20–29 or F30–39 diagnoses) at CAMHS increased the risk of AMHS utilization (see Table 1).

Finally, children and adolescents diagnosed with HD at CAMHS and treated with pharmacological or combined treatment were significantly more likely to seek mental health treatment at AMHS than children and adolescents with HD diagnosed at CAMHS who were not on pharmacological or combined treatment (OR (95% CI) = 1,75 (1,59–1,92); FET *P* < 0.001, and OR (95%  CI) = 1,27 (1,16–1,39); FET *P* < 0.001, resp.). Conversely, psychotherapy was associated with decreased risk of use at AMHS of those children diagnosed with HD at CAMHS (OR (95% CI) = 0,67 (0,61–0,74), FET *P* < 0.001).

## 4. Discussion

Our results show that older age of first diagnosis at CAMHS, female gender, comorbidity between HD and other psychiatric diagnoses at CAMHS, and either pharmacological or combined treatment of HD at CAMHS are factors associated with an increased likelihood of followup at AMHS. These results suggest that the previously mentioned factors could be markers of a worse prognosis in subjects diagnosed with HD at CAMHS.

The prevalence of HD in our sample (7.1%) was higher than the prevalence of HD reported in some community samples [21], but similar to the 6.5% lifetime administrative prevalence rate reported by Döpfner et al. [7]. Other authors have also reported similar figures to those reported here. In an epidemiological study carried out in Rochester, the authors found a cumulative incidence of ADHD in the elementary and secondary school children of 7.5% [22]. In the US National Health Interview Survey, the authors reported a prevalence of 6.7% [12]. In addition, few preschool children were diagnosed with HD in the present study. This is not surprising because pre-school children are not usually referred for treatment [11], and this is also in accordance with the literature [9]. In this study, the authors reported a median age for diagnosis of ADHD of 12 years.

Consistent with the available literature, patients diagnosed with HD at CAMHS were less likely to use AMHS than patients without HD at CAMHS. Although it is widely recognized that ADHD and HD persist into adulthood, less than 20% of adults with hyperactivity are diagnosed or treated [23]. This figure is pretty similar to the 18.7% of patients with HD who were followedup in AMHS reported here. Several reasons may explain why subjects diagnosed with HD at CAMHS are less likely to be followedup in AMHS than subjects without HD at CAMHS. First, although symptoms of inattention, impulsivity, and hyperactivity present differently in adulthood, current diagnostic criteria are geared toward symptom identification during childhood [6, 24, 25]. Second, many adults with hyperactivity are seen by mental health practitioners who are not familiar with the adult presentation of the disorder or are reluctant to diagnose ADHD/HD in adults [1]. Third, symptoms of inattention decline at a modest rate while those of hyperactivity and impulsivity remit much more abruptly [6, 26, 27]. Fourth, children with HD may receive different diagnoses (heterotypic continuities) in adulthood. For instance, some cases of adult ADHD are unrecognized and misdiagnosed with conditions such as borderline personality disorder, atypical depression, or cyclothymia [18]. Fifth, some children with HD may have a better outcome than others, being therefore less likely to use AMHS [6]. Sixth, children with ADHD are more likely to have at least one parent exhibiting noteworthy features of ADHD, if not the full diagnosis, than would be expected by chance [28]. Thus, these families might have greater difficulties following through on treatment and hyperactive children might be less likely to seek services upon reaching adulthood. Finally, there might be inconsistencies in the referral process.

Children diagnosed with HD in CAMHS were more likely followedup at AMHS if first diagnosed at an older age and being of female gender. Kim-Cohen et al. [2] reported that continuities between juvenile mental disorders and mental disorders in adulthood are stronger when diagnoses are made after the age of 15. However, other authors have reported that age is not a critical factor for prediction of persistence of HD [16]. The importance of gender is particularly interesting and might point to differential sex-related etiology in this neurodevelopmental disorder. Furthermore, hyperactivity is frequently a “hidden disorder” in girls [29]. It is well established that the prevalence of ADHD and HD is higher in boys [11, 14]. In addition, more boys than girls with hyperactivity receive treatment [30], which may worsen the prognosis of HD in women. This is unfortunate because hyperactive girls do not differ from boys in terms of response to stimulants [31]. Furthermore, concurrent comorbidity and continuity of mental disorders, including ADHD/HD, are more apparent in girls than in boys at least in the transition from childhood to adolescence [32]. In addition, women are overrepresented in adult samples of hyperactive disorders [16] and seek treatment for hyperactivity more frequently than men [9]. Another explanation is that women diagnosed with childhood HD might suffer from a more severe subtype than men in our study. Finally, as suggested by Kessler et al. [16], it is also possible that, as antisocial behavior is more frequent in hyperactive men, more men than women enter jail and are no longer ascertainable in either epidemiologic or clinical studies. Finally, cultural factors might also contribute to gender differences in service seeking.

Patients diagnosed with HD and concurrent comorbidity were also more likely followed up at AMHS. Comorbidity is a distinct feature of ADHD and HD [6]. Subjects diagnosed with ADHD and comorbid conditions during childhood are more likely diagnosed with ADHD (homotypic continuities) and other psychiatric disorders (heterotypic continuities) in the adulthood [33]. Here, it is important to quote that the ICD-10 exclude the diagnosis of HD in the presence of some comorbid diagnoses such as mood or psychotic disorders. This exclusion criterion might explain why we found a low comorbidity rate of affective and psychotic disorders in patients diagnosed with HD at CAMHS. However, the risk of utilization of AMHS was particularly high for those children and adolescents presenting with HD and comorbid affective disorders (F30–39; ICD-10) and schizophrenia, schizotypal and delusional disorders (F20–29; ICD-10) at CAMHS, thus suggesting that children with HD and comorbid mood and psychotic disorders have a poorer outcome than hyperactive children without such a comorbidity, and underscores the need for treatment guidelines to address this issue. Our findings are in accordance with the literature [6]. For instance, children diagnosed with both schizophrenia and hyperactivity have a poorer response to medication and poorer outcome than those diagnosed with schizophrenia alone [10].

Finally, subjects with HD who received pharmacological treatment at CAMHS were more likely to seek treatment at AMHS than those not receiving medication for the HD at CAMHS. Kessler et al. [16] reported that treatment was associated with persistence in the adulthood. These authors suggested that treatment might be interpreted as a “proxy of severity,” and we concur. Children diagnosed with milder forms of HD at CAMHS might be more likely to receive psychological treatment, thus being less likely to seek treatment at AMHS. This might be interpreted either as an example of the effectiveness of psychological treatment in HD, or as a confirmation that psychological treatment is used in less severe HD cases with a better prognosis.

### 4.1. Strengths and Limitations

The major strength of the present study is that, to our knowledge, this is the largest naturalistic study of transition from CAMHS to AMHS in children and adolescents with HD. Nevertheless, our results should be interpreted in the context of some limitations. First, ICD-10 diagnoses were clinical, nonstandardized diagnoses. However, research and clinical definitions of ADHD and HD rely on reported symptoms and diagnosis is usually made on the basis of clinical impressions [34]. Indeed, rating scales, although useful in documenting hyperactive symptoms as well as the response to treatments, should never be used for diagnosis without careful clinical confirmation [6, 35]. Second, we cannot reject the possibility that our treatment sample was biased due to selection effects (Berkson's bias) [11]. However, our sample size, and the fact that diagnoses were made by several professionals throughout a long period of time, makes this possibility unlikely. Furthermore, it is possible that some patients followed alternative pathways of treatment. Because most Spaniards receive mental health care in public services, this possibility is, however, unlikely [20]. Finally, we used some literature on ADHD to back some of our comments on HD. This was due to the comparatively larger literature on ADHD than on HD. Otherwise, it could have been difficult to put into context some of our findings. Nevertheless, even if DSM-IV ADHDs identify a broader group of hyperactive children than those identified by the ICD-10 HD, there is substantial overlap between both diagnoses [36].

## 5. Conclusions

An older age of first diagnosis, female gender, comorbidity, and pharmacological treatment at CAMHS might be markers of a worse prognosis in subjects diagnosed with HD at CAMHS. Results from the present study might contribute to a reduction in the economic burden of HD by targeting those children with an increased risk of AMHS utilization.

## Figures and Tables

**Table 1:** Factors predicting followup at AMHS of adults diagnosed with hyperkinetic disorder at CAMHS.

	Wald	df	OR (95% CI)	*P* values
Children* (6–12 years old)	4.150	1	1.643 (1.019–2.648)	0.042
Adolescents* (13–17 years old)	29.725	1	3.888 (2.386–6.335)	0.001
Gender (female)	5.455	1	1.414 (1.057–1.893)	0.020
F10–F19 (mental and behavioural disorders due to psychoactive drugs)	8.879	1	2.479 (1.364–4.504)	0.003
F20–F29 (schizophrenia, schizotypal and delusional disorders)	21.325	1	4.351 (2.331–8.123)	<0.001
F30–F39 (mood (affective) disorders)	53.835	1	5.180 (3.338–8.038)	<0.001
F40–F48 (neurotic, stress-related and somatoform disorders)	61.856	1	2.688 (2.101–3.439)	<0.001
F70–F79 (mental retardation)	35.956	1	3.075 (2.130–4.438)	<0.001

*Compared to preschool children. OR: odds ratio; 95% CI: 95% confidence interval.
